# Glacial refugia and postglacial expansion of the alpine–prealpine plant species *Polygala chamaebuxus*

**DOI:** 10.1002/ece3.2515

**Published:** 2016-10-07

**Authors:** Tobias Windmaißer, Stefan Kattari, Günther Heubl, Christoph Reisch

**Affiliations:** ^1^ Institute of Plant Sciences University of Regensburg Regensburg Germany; ^2^ Systematic Botany and Mycology Department Biology I GeoBio‐Center LMU Ludwig‐Maximilians‐University Munich Germany

**Keywords:** AFLP, genetic variation, glacial relict, phylogeography, *Polygala chamaebuxus*

## Abstract

The shrubby milkwort (*Polygala chamaebuxus* L.) is widely distributed in the Alps, but occurs also in the lower mountain ranges of Central Europe such as the Franconian Jura or the Bohemian uplands. Populations in these regions may either originate from glacial survival or from postglacial recolonization. In this study, we analyzed 30 populations of *P. chamaebuxus* from the whole distribution range using AFLP (Amplified Fragment Length Polymorphism) analysis to identify glacial refugia and to illuminate the origin of *P. chamaebuxus* in the lower mountain ranges of Central Europe. Genetic variation and the number of rare fragments within populations were highest in populations from the central part of the distribution range, especially in the Southern Alps (from the Tessin Alps and the Prealps of Lugano to the Triglav Massiv) and in the middle part of the northern Alps. These regions may have served, in accordance with previous studies, as long‐term refugia for the glacial survival of the species. The geographic pattern of genetic variation, as revealed by analysis of molecular variance, Bayesian cluster analysis and a PopGraph genetic network was, however, only weak. Instead of postglacial recolonization from only few long‐term refugia, which would have resulted in deeper genetic splits within the data set, broad waves of postglacial expansion from several short‐term isolated populations in the center to the actual periphery of the distribution range seem to be the scenario explaining the observed pattern of genetic variation most likely. The populations from the lower mountain ranges in Central Europe were more closely related to the populations from the southwestern and northern than from the nearby eastern Alps. Although glacial survival in the Bohemian uplands cannot fully be excluded, *P. chamaebuxus* seems to have immigrated postglacially from the southwestern or central‐northern parts of the Alps into these regions during the expansion of the pine forests in the early Holocene.

## 1 Introduction

The distribution ranges of many plant species were strongly shifted during Quaternary due to rapid and extensive changes in temperature and precipitation which caused multiple events of extinction, isolation, and recolonization (Habel, Drees, Schmitt, & Assmann, 2010). The impact of these climatic changes on the distribution ranges and the genetic structure of plant species can be detected even today and stimulated phylogeographic research (Hewitt, 1996; Kadereit, Griebeler, & Comes, 2004).

The European Alps played an important role in the course of this process as its mountain ranges acted both as refugium throughout several glacial cycles and barriers for range shifts (Brochmann, Gabrielsen, Nordal, Landvik, & Elven, 2003; Taberlet, Fumagalli, Wust‐Saucy, & Cosson, 1998; Tribsch & Schönswetter, 2003). The glacial and postglacial history of numerous high‐alpine and arctic–alpine plant species has been extensively investigated during the last two decades (Eidesen et al., 2013; Paun, Schönswetter, Winkler, Consortium, & Tribsch, 2008; Ronikier, Schneeweis, & Schönswetter, 2012; Stehlik, Blattner, Holderegger, & Bachmann, 2002; Winkler et al., 2012). In many cases, the intraspecific genetic pattern indicated multiple refugia in certain areas throughout the Alps (Schönswetter, Paun, Tribsch, & Niklfeld, 2003; Schönswetter, Tribsch, Stehlik, & Niklfeld, 2004). Bringing together geographic, palaeo‐environmental, and genetic data allowed the general identification of glacial refugia for high‐alpine plant species (Comes & Kadereit, 2003; Mráz et al., 2007).

However, the ecological requirements of plant species have a strong impact on their glacial and postglacial history and different hypotheses about the migration and survival of plant species during Quaternary can therefore be proposed for species with different ecological preferences (Holderegger & Thiel‐Egenter, 2009; Vargas, 2003). Many temperate species, originally occurring in Central Europe, became extinct during the Quaternary ice ages and retreated to southern refugia and survived glacial maxima. In contrast, high‐alpine species even persisted in central refugia on ice‐free mountain tops, so called “nunataks.” Less cold resistant alpine species survived either in refugia at the periphery of the Alps or may have migrated to lowland areas.

Knowledge about the vegetation of these lowlands between the Scandinavian and Alpine ice sheet in Central Europe during glaciation is yet scarce. There are stratigraphic records of pollen and macrofossils for *Salix herbacea*,* Betula nana*,* Dryas octopetala,* or *Koeningia islandica*, whereas dwarf shrubs counting among Ericaceae played an unexpectedly subordinate role (Burga, Klötzli, & Grabherr, 2004; Lang, 1994). Clear evidence for the survival of alpine plant species in the prealpine region exists for *Minuartia biflora* (Schönswetter, Popp, & Brochmann, 2006), but several other species were also supposed to have survived in Central Europe (Bauert, Kälin, Baltisberger, & Edwards, 1998; Holderegger, Stehlik, & Abbott, 2002; Reisch, 2008; Reisch, Poschlod, & Wingender, 2003). Cryptic refugia in Central Europe have previously been postulated especially for forest herbs, grasses, or shrubs such as *Cicerbita alpina* (Michl et al., 2010), *Polygonatum verticillatum* (Kramp, Huck, Niketić, Tomović, & Schmitt, 2009), *Cyclamen purpurascens* (Slovák, Kučera, Turis, & Zozomová‐Lihová, 2012), *Melica nutans* (Tyler, 2002), *Hordelymus europaeus* (Dvořáková, Fér, & Marhold, 2010), or *Rosa pendulina* (Fér, Vašák, Vojta, & Marhold, 2007). This must, however, not necessarily be the case, as postglacial recolonization of the Alps from peripheral refugia may also have included migration to the lower mountain ranges of Central Europe. Central European lowland populations of plant species being mainly distributed in the Alps may therefore be either the result of glacial survival or of postglacial immigration.

The shrubby milkwort (*Polygala chamaebuxus*) is an endemic European species with a remarkably broad ecological niche and a wide distribution range including the Alps but also Central European mountain ranges like the Franconian Jura or the Bohemian uplands. There, it occurs mainly in pine forests and on rocky mountain slopes. In the study presented here, we tried to illuminate the origin of the species in these lower mountain regions. More specifically, our aim was (i) to identify glacial refugia of *P. chamaebuxus* and (ii) to analyze whether the populations of the species in the low mountain ranges can be attributed rather to glacial survival or to postglacial immigration.

## 2 Materials and methods

### 2.1 Species description

*Polygala chamaebuxus* L. belongs to the small subgen. *Chamaebuxus* (DC) Schb. which includes five perennial species of shrubs or dwarf shrubs, with alternate, subcoriaceous leaves, flowers with a crest on the keel, winged capsule, and carunculated seeds. Actually four species of this lineage are known from Europe: *P. chamaebuxus* L. (widespread throughout the Alps), *P. vayredae* Costa (endemic to Catalonia, Spain), *P. balansae* Coss., and *P. webbiana* Coss. (distributed in Morocco), both taxa recently reported from southern Spain (Calvo, Hantson, & Paiva, 2014; Lorite, Peňas, Benito, Caňadas, & Valle, 2010). In addition, the subgenus includes one species which is restricted to northwestern Africa: *P. munbyana* Boiss. & Reut.

Based on karyological and palynological studies (Merxmüller & Heubl, 1983), it was suggested that *P. munbyana* (2*n *= 14) belongs to the diploid level, *P. webbiana, P. balansae,* and *P. vayredae* are tetraploids with 2*n *= 28, whereas hyperhexaploidy (2*n *= 44) was found in *P. chamaebuxus*. Karyotype analysis revealed that *P. chamaebuxus* developed most probably by autopolyploidy from *P. vayredae* or the African *P. webbiana* or by allopolyploidy of these species. The evolution of the group concerned seems to have taken place in the southwestern Mediterranean and to have continued on the Iberian way as far as the Alps and Central Europe (Merxmüller & Heubl, 1983).

In contrast to the Iberian taxa which are narrow endemics, *P. chamaebuxus* L. has the largest and northernmost distribution range of all members. It occurs in the Alps, the northern Apennine, the northern parts of the Dinaric Mountains, and in parts of the prealpine moraine landscape as well as some in low mountain ranges like such as Jurassic mountains, the Bavarian Forest, the Fichtelgebirge, and the Bohemian uplands (Sebald, Seybold, Philippi, & Wörz, 1998). A white flowered form of *P. chamaebuxus* occurs, most probably, over the whole distribution range, whereas a red flowered form (var *grandiflora* Gaudin; var *rhodoptera* Ball) can only be found in the cantons of Graubünden and Tessin and down the Apennine (Meusel, Jäger, Rauschert, & Weinert, 1978).

*Polygala chamaebuxus* is a 5‐ to 30‐cm‐high dwarf shrub. Full flowering occurs in spring and early summer. The species is, like the closely related species *P. vayredae* (Castro, Loureiro, Ferrero, Silveira, & Navarro, 2013; Castro, Silveira, & Navarro, 2008), insect‐pollinated, allogamous, and self‐incompatible (Hegi, 1986; Jauch, 1917). *Polygala chamaebuxus* exhibits a broad ecological range. It grows in open forests, mainly pine woods, among rocks and mountain slopes. According to phytosociological classification, this taxon is together with *Erica carnea* a characteristic element of the order Erico‐Pinetalia. In the Alps, it reaches up to 2,650 m above sea level in Graubünden (Braun‐Blanquet & Rübel, 1932), and at Monte Baldo, it can be found from 80 m above sea level up to 2,100 m altitude (Prosser, Bertolli, & Festi, 2009). It grows predominantly on calcareous soil types but also some populations on more acidic soils have been reported. *Polygala chamaebuxus* is a medium shade plant and the light supply seems to be one of the most important factors, which is strongly influenced by the surrounding vegetation (Gauckler, 1938). Therefore, it occurs predominantly in sparse pine woods, dry oak forests, as well as on calcareous low‐nutrient meadows (Sebald et al., 1998).

### 2.2 Study design and sampling of plant material

**Figure 1:**
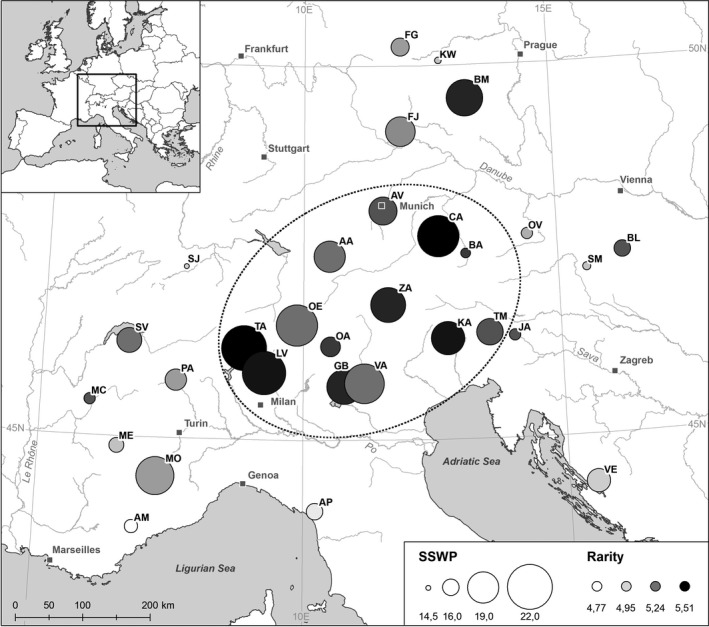
Genetic variation within the studied populations, measured as AMOVA‐derived SSWP/*n *− 1 values (SSWP) and rarity index (DW). Circle diameter and color indicate the degree of genetic variation. The dotted line marks the area with high levels of genetic variation and rarity within populations in the center of the distribution range

For the study presented here, plant material was sampled from 30 populations (Table 1, Figure 1) covering continuously almost the entire range of *P. chamaebuxus*. When possible, within populations, ten samples were taken with a minimum distance of ten meters following a transect to avoid double sampling of the same individual.

**Table 1:** Geographic location of the studied *Polygala chamaebuxus* populations with number, population code, name of the location as well as geographic longitude (Long.), latitude (Lat.) and altitude. Populations were numbered across the distribution range from west to east and north to south

Nr.	Code	Location	Long. (E)	Lat. (N)	Altitude (m)
01	FG	Fichtelgebirge	11,97371	50,25392	524
02	KW	Slavkowsky les	12,75008	50,06559	807
03	BM	Bohemian Massiv	13,27324	49,55553	496
04	FJ	Fränkischer Jura	11,94680	49,12638	387
05	AV	Alpenvorland	11,56941	48,06784	563
06	CA	Chiemgauer Alpen	12,65713	47,71825	711
07	OV	Oberösterreichische Voralpen	14,41594	47,71413	791
08	SJ	Schweizer Jura	7,700333	47,30297	547
09	AA	Allgäuer Alpen	10,50837	47,46366	1,186
10	BA	Berchtesgadener Alpen	13,18686	47,48139	641
11	SM	Steiermark	15,55841	47,23277	575
12	BL	Burgenland	16,27630	47,43672	774
13	OE	Oberengadin	9,875055	46,54116	1,793
14	ZA	Zillertaler Alpen	11,64729	46,81123	1,120
15	SV	Savoyen/ Chablais	6,641444	46,28488	1,237
16	TA	Tessiner Alpen	8,858833	46,22941	919
17	OA	Ortler Alpen	10,52377	46,25705	1,387
18	KA	Karnische Alpen	12,79445	46,35128	1,304
19	TM	Triglav Massiv	13,60812	46,41775	986
20	JA	Julische Alpen	14,09105	46,36751	500
21	PA	Penninische Alpen	7,566597	45,78042	1,555
22	LV	Luganer Voralpen	9,24875	45,90025	1,282
23	GB	Gardasee Mountains	10,78505	45,71894	257
24	VA	Vizentiner Alpen	11,17294	45,76063	1,174
25	MC	Massif de la Chartreuse	5,940111	45,47738	831
26	ME	Massif des Écrins	6,493944	44,87375	1,438
27	MO	Massif dell′Oronaye	7,240055	44,48855	853
28	AP	Apennin	10,22541	44,05240	1,353
29	VE	Velebit	15,52575	44,35925	1,457
30	AM	Alpes maritimes	6,836888	43,79827	1,193

### 2.3 AFLP analysis

For AFLPs, the DNA was extracted from the dried sampling material following the CTAB protocol from Rogers and Bendich (1994) adapted by Reisch and Kellermeier (2007). After photometrical measurement of the concentration, solutions were diluted with water to 7.8 ng/μl and were subsequently used for AFLPs, which were conducted in accordance with the protocol of Beckmann Coulter as described before (Bylebyl, Poschlod, & Reisch, 2008; Reisch, 2008).

After an initial screening of 30 primer combinations, three of them were chosen for the subsequent selective PCR reaction using labeled EcoRI primers (M‐CAC/D2‐E‐AGC, M‐CAA/D3‐E‐ACG, M‐CTT/D4‐E‐ACT, Beckman Coulter). The resulting products were diluted twofold (D2) and fivefold (D4) with 1× TE_0.1_ buffer for AFLP, while the D3 products remained undiluted. Subsequently, 5 μl of each of the diluted PCR products of a given sample was pooled and added to a mixture of 2 μl sodium acetate (3 mol/L, pH 5.2), 2 μl Na 2 EDTA (100 mmol/L, pH 8), and 1 μl glycogen (Roche). DNA was precipitated in a 1.5‐ml tube by adding 60 μl of 96% ethanol (−20°C) and 20‐min centrifugation at 14,000 × g at 4°C. The supernatant was poured off, and the pellet was washed by adding 200 μl 76% ethanol (−20°C) and centrifugation at the latter conditions.

The pelleted DNA was vacuum dried in a vacuum concentrator. Subsequently, the pellet was dissolved in a mixture of 24.8 μl Sample Loading Solution (SLS, Beckman Coulter) and 0.2 μl CEQ Size Standard 400 (Beckman Coulter) and subsequently selective PCR products were separated by capillary gel electrophoresis on an automated sequencer (CEQ 8000, Beckmann Coulter).

Results were examined using the CEQ 8000 software (Beckman Coulter) and analyzed using the software Bionumerics 6.6 (Applied Maths, Kortrijk, Belgium). In order to assess the reproducibility of the scored fragments, about 10% (29 samples) of all analyzed samples were repeated and the genotyping error rate (Bonin et al., 2004) was estimated, which was 4.8%.

### 2.4 Statistical analysis

Using the resulting binary matrix, genetic variation within populations was determined applying the program PopGene 1.32 (Yeh, Yang, Boyles, Ye, & Mao, 1997) as percentage of polymorphic bands PB and Nei's gene diversity *H* = 1 − Σ(*p*_i_)². Additionally, we calculated rarity as frequency down weighted markers (DW) for each population (Schönswetter & Tribsch, 2005) with AFLPdat in R (Ehrich, 2006). Therefore, we randomly chose eight individuals per population in five iterations.

A Bayesian cluster analysis using 10,000 Markov chain Monte Carlo (MCMC) simulations was computed with 20 iterations per *K* = 1–31 and a burning period of 10,000 with the software Structure 2.3.3 (Pritchard, Stephens, & Donelly, 2000). The most probable number of classes was calculated (Evanno, Regnaut, & Goudet, 2005), and the mean probability of the individuals of each population to be assigned to the respective classes was calculated over all 20 repeats for the most probable number of classes.

Furthermore, a nonhierarchical AMOVA was carried out with GenAlEx 6.41 (Peakall & Smouse, 2006) based on pairwise Euclidian distances to assess the variation within and among populations. This also yielded pairwise PhiPT values as well as the SSWP value (sum of squares within population) for each population. Dividing the latter value through the number of individuals reduced by one, provided the sample size‐independent measure of variation SSWP/(*n *− 1).

A Mantel test was performed to analyze whether the genetic distances and the geographic distances between populations were correlated (Mantel, 1967).

Finally, we used PopGraph (Dyer & Nason, 2004) to calculate the conditional graph distance derived from population networks (Dyer, Nason, & Garrick, 2010). Analyses were performed with Genetic Studio (http://dyerlab.bio.vcu.edu/software.html). PopGraph is free of a priori assumptions about population geographic arrangements and uses a graph theoretical approach to determine the minimum set of edges (connections) that sufficiently explain the total among‐population covariance structure of all of the populations (Dyer & Nason, 2004).

## 3 Results

AFLP fingerprinting of 296 individuals resulted in 174 fragments of which 94.6% were polymorphic. The percentage of polymorphic loci within populations (PB) ranged from 43.7 to 67.2 with a mean of 53.1 (Table 2). Nei's gene diversity (*H*) within the studied populations varied between 0.16 and 0.26 with an average of 0.21, whereas the AMOVA‐derived diversity measurement SSWP/(*n *− 1) ranged from 14.5 to 22.0 with a mean of 17.5. The rarity index (DW) showed only little differences between populations and ranged from 4.77 to 5.51 with an average of 5.21. However, rarity was highest in populations with high levels of Nei's gene diversity as revealed by correlation analysis using Spearman's rank correlation coefficient (r = .61, *p* < .001). Genetic variation within populations and the rarity index were highest in populations from the central part of the distribution range (Figure 1), especially in the Southern Alps from the Tessin Alps (population TA) to the Triglav Massiv (population TM). This applies particularly to the populations in the Tessin Alps and the Prealps of Lugano (population LV). Another center of genetic variation was located in the middle part of the northern Alps (population CA). Genetic variation generally decreased toward the periphery of the distribution range. Except for two populations from the Southern Alps in France (Population MO) and the Bohemian Massif (population BM), most populations in the eastern Alps, western Alps, the Apennines, or the lower mountain ranges in the northern part of the distribution area showed values of genetic variation and rarity below average.

**Table 2:** Genetic variation of the studied *Polygala chamaebuxus* populations with number, population code, and name of the location. For each population, the percentage of polymorphic loci (PB), Nei's gene diversity (*H*), the AMOVA‐derived SSWP/*n *− 1 (SSWP), and the rarity index (DW) are listed. Populations were numbered across the distribution range from west to east and north to south

Nr.	Code	Location	*n*	PB	*H*	SSWP	DW
01	FG	Fichtelgebirge	10	51.2	0.20	16.2	5.14
02	KW	Slavkowsky les	10	44.8	0.18	14.8	5.01
03	BM	Bohemian Massiv	10	60.3	0.24	19.4	5.39
04	FJ	Franconian Jura	10	56.3	0.22	18.8	5.16
05	AV	Prealps	10	55.8	0.22	18.4	5.29
06	CA	Chiemgauer Alps	10	59.8	0.23	20.3	5.50
07	OV	Oberösterr. Prealps	10	48.9	0.20	15.2	5.07
08	SJ	Swiss Jura	10	43.7	0.16	14.5	4.95
09	AA	Allgäuer Alps	10	55.8	0.22	19.0	5.23
10	BA	Berchtesgadner Alps	8	44.3	0.17	15.0	5.31
11	SM	Steiermark	10	46.0	0.18	14.9	5.03
12	BL	Burgenland	10	49.4	0.19	16.0	5.26
13	OE	Oberengadin	10	61.5	0.25	20.3	5.23
14	ZA	Zillertaler Alps	10	58.1	0.23	19.3	5.37
15	SV	Savoyen/ Chablais	10	51.2	0.20	17.1	5.24
16	TA	Tessin Alps	10	67.2	0.26	22.0	5.51
17	OA	Ortler Alps	9	46.6	0.19	16.3	5.30
18	KA	Carnic Alps	10	58.1	0.23	19.1	5.46
19	TM	Triglav Massiv	10	53.5	0.21	17.8	5.27
20	JA	Julic Alps	10	47.1	0.18	15.2	5.28
21	PA	Penninic Alps	10	49.4	0.20	16.6	5.11
22	LV	Lugano Prealps	10	62.1	0.25	21.6	5.47
23	GB	Gardasee Mountains	10	56.9	0.23	19.3	5.37
24	VA	Vizentiner Alps	10	62.6	0.26	20.2	5.24
25	MC	Massif de la Chartreuse	10	48.3	0.19	15.2	5.29
26	ME	Massif des Écrins	10	49.4	0.20	15.6	5.04
27	MO	Massif dell′Oronaye	10	59.2	0.24	19.5	5.11
28	AP	Apennin	9	47.7	0.19	16.0	4.89
29	VE	Velebit	10	53.5	0.19	16.8	4.93
30	AM	Alpes maritimes	10	46.0	0.18	15.4	4.77
	Mean			53.1	0.21	17.5	5.2
	±SE			6.4	0.03	2.2	0.2

**Figure 2:**
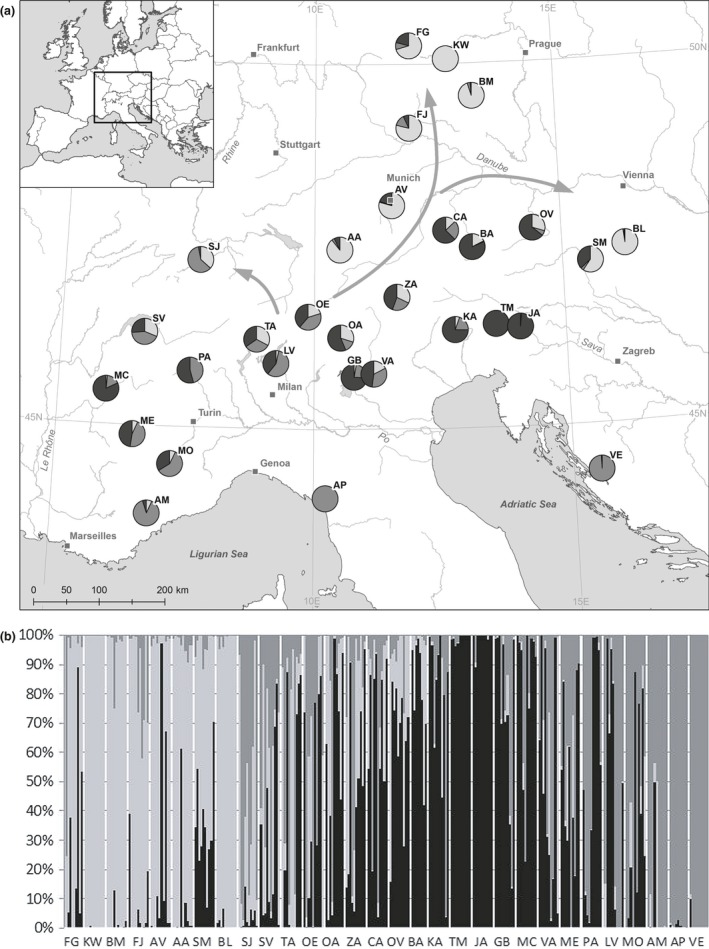
Assignment of the studied individuals to the three groups (white, bright gray, or black) detected in the Bayesian cluster analysis as cumulated percentages from the STRUCTURE analysis. Arrows indicate possible postglacial migration routes

The Bayesian cluster analysis revealed only a comparatively weak geographic pattern of genetic variation. Following the analysis, the data set consisted most likely of three groups (Figure 2a,b), although none of populations was completely assigned to only one group. However, populations from the northeastern part of the distribution range were mainly assigned to one group, while the populations from the southwest and the southeast were more frequently classified in two other groups.

In a nonhierarchical analysis of molecular variance (AMOVA), only 16.5% of the total genetic variation was found among all populations while 83.5% were detected within populations (Table 3). The overall Φ_PT_ was therefore 0.17. Variation between the groups detected in the Bayesian cluster analysis was significant but with only 3% very low. Similarly, molecular variance between the northeastern group on the one hand and the southeastern and southwestern group on the other hand was only 4% and, therefore, also very low. A Mantel test showed a significant correlation of the genetic variation between populations obtained from the AMOVA (Φ_PT_) and the respective geographic distance between populations (r* *=* *.570, *p *<* *.001).

**Table 3:** Results of the conducted analyses of molecular variance (AMOVA). We calculated variation between all populations (1), between the three groups derived from the Bayesian cluster analysis (2) between the northern group and the western (3) and eastern group (4)

Level of variation	*df*	SS	MS	VC	VC%
(1) All populations					
Among populations	29	1,498.2	51.7	3.46	16.5
Within populations	266	4,668.4	17.6	17.55	83.5
(2): [SW]–[E]–[N]					
Among regions	2	204.6	102.3	0.6	3.0
Among populations within regions	27	1,293.6	47.9	3.1	15.0
Within populations	266	4,668.4	17.6	17.1	83.0
(3): [SW]–[N]					
Among regions	1	114.7	114.7	0.9	4.0
Among populations within regions	18	852.2	47.3	3.0	14.0
Within populations	178	3,151.6	17.7	17.7	82.0
(4): [E]–[N]					
Among regions	1	95.7	95.7	0.8	4.0
Among populations within regions	13	584.8	44.9	2.8	13.0
Within populations	133	2,305.5	17.3	17.3	83.0

SW, southwestern group; E, eastern group; N, northern group; *df*, degrees of freedom; SS, sum of squares; MS, means squares; VC, variance components; VC, proportion of variance in %. All calculations were significant at *p* < .001.

**Figure 3:**
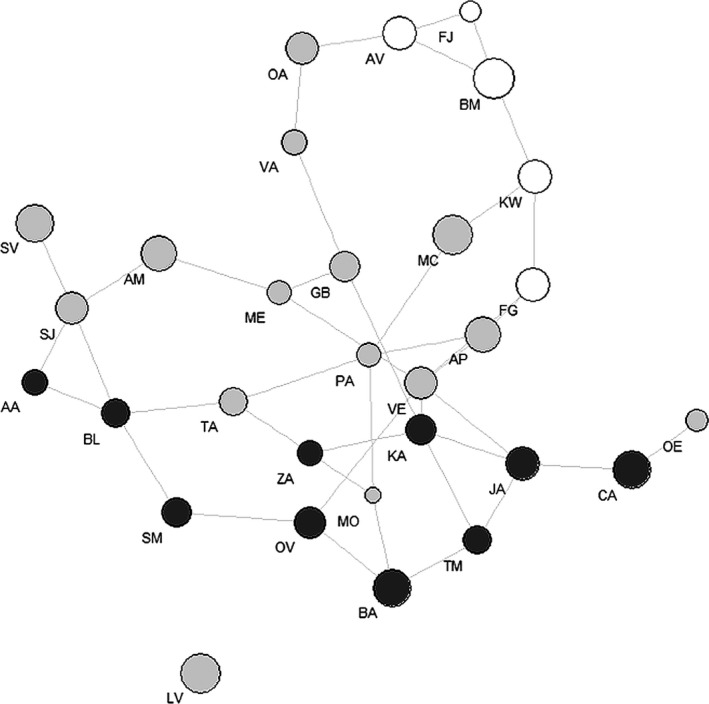
PopGraph genetic network for all studied populations. Circle size reflects the levels of genetic variation within populations. Lines show component of genetic variation between populations due to connecting nodes. Letters within circles indicate the populations following Table 1. Populations from the lower mountain ranges in Central Europe are displayed in white, populations from the western part of the distribution range in light gray, and populations from the eastern part in dark gray

In the PopGraph genetic, network populations were highly interconnected (Figure 3). However, the populations from the northern group detected in the Bayesian cluster analysis were more closely related to the populations from the southwestern than to the populations from the southeastern group. One of the most variable populations also containing a higher number of rare fragments (population LV) was completely separated from the network.

## 4 Discussion

### 4.1 Genetic variation of *Polygala chamaebuxus* in the context of life history traits

It has already been demonstrated that life history traits have a strong impact on genetic variation within and between populations. In particular, life span, frequency, and mating system are of outstanding importance for genetic variation (Nybom, 2004; Reisch & Bernhardt‐Römermann, 2014). The genetic variation within populations of *P. chamaebuxus* observed in our study (*H* = 0.21) was comparable to the variation recently reported for other long‐lived, common, and outcrossing plant species (*H* = 0.20) using AFLPs (Reisch & Bernhardt‐Römermann, 2014). The results of our study match, from this point of view, the findings of the preceding reviews.

In contrast to our expectations, we observed, however, only a low level of genetic variation between populations of *P. chamaebuxus*. Previously, for long‐lived, common, and outcrossing plant species, a mean Φ_PT_ of 0.20–0.34 was reported (Reisch & Bernhardt‐Römermann, 2014). As genetic variation depends on life history traits, the comparison of single species with differing traits is always delicate. Nevertheless, many alpine species exhibited even higher levels of genetic differentiation (Schönswetter et al., 2004; Vogler & Reisch, 2013). With a Φ_PT_ of only 0.17 between all populations across the whole distribution range, *P. chamaebuxus* exhibited only a weak geographic pattern of genetic variation. This suggests a comparatively short period of isolation during the glaciations and rather broad waves of postglacial recolonization as discussed more detailed below.

### 4.2 Glacial refugia and postglacial recolonization

Following our data, especially the high level of rarity, suggests long‐term survival of *P. chamaebuxus* in the Southern Alps between Switzerland and Italy. This area has already been identified as refugium for other calcicolous, subalpine to lower alpine plant species in previous studies (Tribsch & Schönswetter, 2003). Another putative refugium of *P. chamaebuxus* has probably been located in the middle part of the northern Alps, where we also observed a higher number of rare fragments. The occurrence of *P. chamaebuxus* along the northern margin of the Alps at least during the last interglacial (Eemian) has been proved by fossil evidence (Murr, 1926; Wettstein, 1892) and previous studies have already postulated glacial refugia at the northern edge of the Alps (Schönswetter, Stehlik, Holderegger, & Tribsch, 2005; Stehlik, 2003), which supports the assumption that *P. chamaebuxus* could have survived glaciations also in this region.

However, our results indicate rather a genetic continuum than deep genetic splits between populations of *P. chamaebuxus*, which may be a sign of a comparatively short period of isolation during the LGM. It is known that the strong glaciations of the Würm glaciation were limited to few periods of extreme cold climate with culmination during the LGM (Veit, 2002). During the climatically warmer interstadial periods, the species might indeed have been distributed widely throughout the Alps. *Polygala chamaebuxus* exhibits a broad ecological range, which allows the species to grow under various climatic conditions and is even considered as cold germinator (Jäger, 2011). *Polygala chamaebuxus* may, for this reason, have been affected not that strongly by the glaciations like other highly specialized species. It is possible that the refugia described above were locations where the species survived most time of the Pleistocene. However, based on the results of the Bayesian cluster analysis, it appears likely that further locally surviving populations in other regions also contributed to the postglacial recolonization after the LGM. The geographic pattern of genetic variation revealed by the Bayesian cluster analysis may therefore reflect not only postglacial recolonization but also gene flow and range expansion from the periods before the LGM, which is also supported by the positive relationship of genetic and geographic distance in the Mantel test. Instead of postglacial recolonization from only few long‐term refugia, which would have resulted in deeper genetic splits within the data set, broad waves of postglacial expansion from multiple populations in the center to the actual periphery of the distribution range seem to be the scenario explaining the observed pattern of genetic variation most likely.

### 4.3 Glacial survival in the lower mountain ranges or not?

The populations of *P. chamaebuxus* in the lower mountains of Central Europe, such as the Jurassic mountains, the Bavarian Forest, the Fichtelgebirge, and the Bohemian uplands, may originate from glacial survival or postglacial immigration. Interestingly, our results provide evidence for both the survival and immigration hypotheses. The number of rare fragments was not conspicuously increased, except for the population from the Bohemian massif, which could in fact indicate long‐term survival in this region. It can therefore not fully be excluded that the species survived glaciations in the Bohemian uplands.

This assumption is supported by previous studies reporting glacial survival of forest‐related plant species in cryptic refugia located in the lower Central European mountain ranges (Kramp et al., 2009; Michl et al., 2010; Slovák et al., 2012; Tyler, 2002), although some studies also revealed ambiguous results (Dvořáková et al., 2010; Fér et al., 2007). Kramp et al. (2009) for example suggested the survival of *Polygonatum verticillatum* in the Tatra and Sudety Mountains. Similarly, it is assumed that the boreo‐montane tall forb *Cicerbita alpina* survived glaciations in sheltered pockets with a humid climate in some parts of Central Europe (Michl et al., 2010) and that *Cyclamen purpurascens* may also have survived glaciations in prealpine northern refugia (Slovák et al., 2012). For the woodland grass *Melica nutans,* several independent “strongly restricted and isolated” refugia in Central Europe have been detected (Tyler, 2002). It is therefore quite possible that *P. chamaebuxus* survived glaciations in the Bohemian massif.

However, we observed no deep genetic split between the Central European populations and populations from other regions. From this point of view, it seems to be likely that most populations spread postglacially to the range periphery and the lower mountains of Central Europe. Founder effects and long‐distance dispersal associated with this expansion may have resulted in the lower levels of genetic variation observed in the more peripheral populations. The probably remnant lineage of the Bohemian massif might have been genetically merged in the expanding wave from the northern Alps.

In the PopGraph genetic network, the populations from the lower mountain regions were more closely related to the populations from the western part than to the populations from the eastern part of the distribution range. This suggests that *P. chamaebuxus* may have immigrated postglacially from the southwestern or central‐northern part of the Alps to the lower mountains of Central Europe. This migration process of *P. chamaebuxus* to the lower mountain regions may be associated with the expansion of pine forests after the last LGM. It is assumed that *Pinus sylvestris* survived glaciations on the Iberian and the Balkan Peninsula (Sinclair, Morman, & Ennos, 1999; Soranzo, Alia, Provan, & Powell, 2000; Wójkiewicz & Wachiowak, 2016). However, cryptic northern refugia have also been postulated for Scots pine (Kinloch, Westfall, & Forrest, 1986; Stewart & Lister, 2001), similar to the herbaceous forest species mentioned above. Whereas the Iberian populations are considered as relicts, Central Europe and Scandinavia were recolonized postglacially from the Balkan (Wójkiewicz & Wachiowak, 2016). From there, pine forests spread in the early postglacial phases and covered large parts of the alpine forelands and Central Europe (Lang, 1994). *Polygala chamaebuxus* is considered as a species typically for these early pine forests (Hardtke & Ihl, 2000) and still occurs today in this type of habitat (Gauckler, 1938). The widely distributed postglacial pine forests seem to have provided well conditions for a broad and continuous co‐migration of *P. chamaebuxus* together with Scots pine toward the north. Migration could already have been started in the Late Glacial from 15,000 BP to 10,000 BP as pine and birch were already present in the Alps and the alpine forelands until about 8,000 BP when the continuous distribution of pine forests ended (Lang, 1994; Veit, 2002).

Similarly, the species seems to have migrated from the center of the distribution range to the eastern and western Alps. In this context, it is a remarkable finding of our study that the population from the Velebit in Croatia was more closely related to the population from the Apennine and westernward populations than to the populations from the nearby southeastern Alps. This observation was also made for *Saxifraga paniculata* in a previous study (Reisch, 2008) and seems to be linked to the desiccation of the Adriatic during glaciation, which seems to have alleviated migration processes.

## Conflict of Interest

None declared.
